# Methyl 9*H*-xanthene-9-carboxyl­ate

**DOI:** 10.1107/S1600536808005990

**Published:** 2008-04-16

**Authors:** Pamela M. Dean, Jelena Turanjanin, Douglas R. MacFarlane

**Affiliations:** a School of Chemistry, Monash University, Wellington Road, Clayton, Victoria 3800, Australia

## Abstract

The title compound, C_15_H_12_O_3_, was obtained unintentionally as the by-product of an attempted recrystallization from methanol of propantheline bromide, an anti­muscarinic drug. The xanthone unit is folded, with a dihedral angle of 24.81 (9)° between the benzene rings. The ester substituent adopts a *trans* staggered conformation, with a C—C—O—C torsion angle of 178.4 (1)°. The mol­ecules pack in distinct layers, facilitated by C—H⋯π and weak π–π ring inter­actions. A weak C—H⋯O inter­action also occurs; however, no classical hydrogen bonding is observed.

## Related literature

For details of the first spectroscopic evidence of the trans­esterification of propantheline bromide by methanol to 9*H*-xanthene-9-carboxylic acid methyl ester, see: Avdovich *et al.* (1986 ▶). For a description of the comparative effectiveness of propantheline bromide for the treatment of neurogenic detrusor overactivity, see: George *et al.* (2007 ▶).
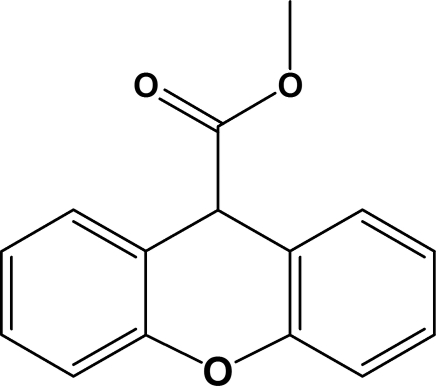


## Experimental

### 

#### Crystal data

C_15_H_12_O_3_
*M* *_r_* = 240.25
Monoclinic, 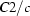

*a* = 25.6601 (16) Å
*b* = 5.7624 (3) Å
*c* = 15.7578 (9) Å
β = 92.933 (4)°
*V* = 2327.0 (2) Å^3^
*Z* = 8
Mo *K*α radiation
μ = 0.10 mm^−1^
*T* = 123 (2) K
0.50 × 0.50 × 0.50 mm

#### Data collection

Bruker Kappa APEXII diffractometer
Absorption correction: multi-scan (*SADABS*; Bruker, 2005 ▶) *T*_min_ = 0.932, *T*_max_ = 0.954
11906 measured reflections
2672 independent reflections
1985 reflections with *I* > 2σ(*I*)
*R*_int_ = 0.050

#### Refinement

*R*[*F* ^2^ > 2σ(*F* ^2^)] = 0.050
*wR*(*F* ^2^) = 0.106
*S* = 1.05
2672 reflections
166 parameters
H-atom parameters constrained
Δρ_max_ = 0.20 e Å^−3^
Δρ_min_ = −0.21 e Å^−3^

### 

Data collection: *APEX2* (Bruker, 2005 ▶); cell refinement: *APEX2*; data reduction: *APEX2*; program(s) used to solve structure: *SHELXS97* (Sheldrick, 2008 ▶); program(s) used to refine structure: *SHELXL97* (Sheldrick, 2008 ▶); molecular graphics: *POV-RAY for Windows* (Persistence of Vision, 1999 ▶); software used to prepare material for publication: *PLATON* (Spek, 2003 ▶).

## Supplementary Material

Crystal structure: contains datablocks I, global. DOI: 10.1107/S1600536808005990/wn2242sup1.cif

Structure factors: contains datablocks I. DOI: 10.1107/S1600536808005990/wn2242Isup2.hkl

Additional supplementary materials:  crystallographic information; 3D view; checkCIF report

## Figures and Tables

**Table 1:** Hydrogen-bond geometry (Å, °)

*D*—H⋯*A*	*D*—H	H⋯*A*	*D*⋯*A*	*D*—H⋯*A*
C15—H15*C*⋯O2^i^	0.98	2.53	3.407 (3)	149
C3—H3⋯*Cg*2^ii^	0.95	2.95	3.668 (2)	133
C11—H11⋯*Cg*1^iii^	0.95	3.18	3.825 (2)	127
C15—H15*B*⋯*Cg*1^iv^	0.98	3.06	3.432 (2)	104
C15—H15*C*⋯*Cg*1^iv^	0.98	3.11	3.432 (2)	101

Symmetry codes: (i) 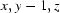; (ii) 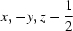; (iii) 
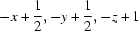
; (iv) 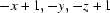. *Cg*1 is the centroid of ring C1–C6; *Cg*2 is the centroid of ring C8–C13.

**Table 2:** Geometrical parameters (Å, °) of the inter-ring π—π interactions

*CgI*	*CgJ*	*Cg*⋯*Cg*	α	Symmetry position of *CgJ*
*Cg*1	*Cg*2	5.590 (1)	59.44	*x*, 1 − *y*, 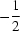 + *z*
*Cg*1	*Cg*2	4.944 (1)	24.81	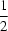 − *x*, 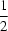 − *y*, 1 − *z*
*Cg*2	*Cg*1	4.863 (1)	59.44	*x*, −*y*, 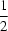 + *z*
*Cg*2	*Cg*2	3.684 (1)	0.03	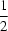 − *x*, 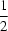 − *y*, 1 − *z*

α is the dihedral angle between planes *I* and *J*, *CgI* is the centroid of plane *I* and *CgJ* the centroid of plane *J*. *Cg*1 is the centroid of ring C1–C6; *Cg*2 is the centroid of ring C8–C13.

**Fig. 1.**
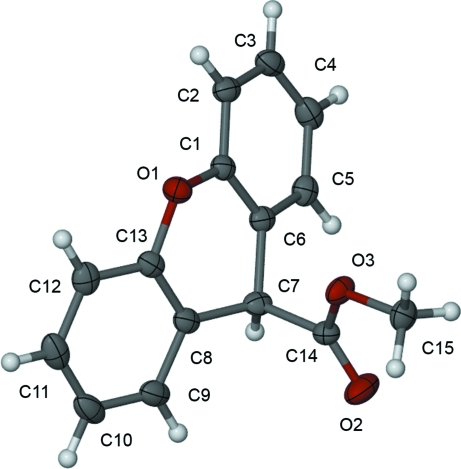
The molecular structure of the title compound, with displacement ellipsoids drawn at the 50% probability level and hydrogen atoms as spheres of arbitrary radius.

**Fig. 2.**
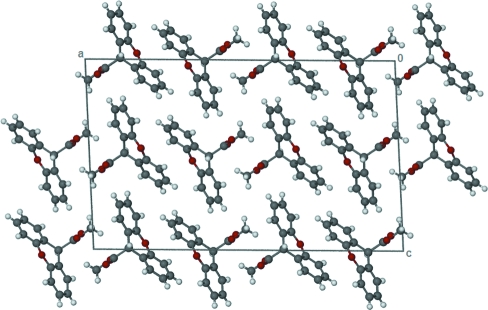
A ball-and-stick representation of the unit-cell contents, viewed down the b axis.

## supplementary crystallographic information

### Comment

It was found that propantheline bromide (George *et al.*, 2007) undergoes facile transesterification by methanol to produce the by-product 9*H*-xanthene-9-carboxylic acid methyl ester (Avdovich *et al.*, 1986). Surprisingly, the structural elucidation of this analogue (Fig. 1) has not been reported in the literature until now. Now the structural determination and analysis is briefly described.

The xanthone unit is bent, with the aromatic planes oriented to each other by an interplanar angle of 24.81 (9)°. The ester substituent adopts a *trans* staggered conformation with a C7—C14—O3—C15 torsion angle of 178.4 (1)°. Additionally, as is typical of an ester, the O3—C14 distance is 1.326 (2) Å and the O3—C15 distance is 1.448 (2) Å, indicating the *sp^2^* hybridization of C14.

The overall packing is shown in Fig. 2. Molecules are related by centres of symmetry, resulting in a head-to-head arrangement, that packs in aromatic and non-aromatic layers lying parallel to the (100) plane. Fig. 2 displays the orientation of the molecules, facilitating the weak C—H···O hydrogen bonding between the methyl and carbonyl groups (distance: C15—H15C···O2^i^ (i = *x*,*y* - 1,*z*) 3.407 (2) Å - see Table 1) and the C—H···π and weak π···π ring interactions (Table 2). A short range contact, 2.683 (2) Å, also occurs between the aromatic C4—H4 and the carbonyl oxygen O2 (distance: C4—H4···O2^ii^ (ii = *x*,1 - *y*,-1/2 + *z*).

### Experimental

The title compound was obtained unintentionally as the product of an attempted recrystallization of propantheline bromide (50 mg) in methanol (2 ml) at room temperature. Crystals resulted after 6 days; these were coated with Paratone N oil (Exxon Chemical Co., TX, USA) immediately after isolation and cooled in a stream of nitrogen vapour on the diffractometer. Melting point: 360.7 K.

### Refinement

All H atoms were observed in difference syntheses and were then placed in geometrically idealized positions and constrained to ride on their parent atoms with C—H distances in the range 0.95–1.00 Å. *U*_iso_(H) = *xU*_eq_(C), where *x* = 1.5 for methyl and 1.2 for all other C atoms.

### Crystal data

**Table:**

C_15_H_12_O_3_	*F*_000_ = 1008
*M**_r_* = 240.25	*D*_x_ = 1.372 Mg m^−^^3^
Monoclinic, *C*2/*c*	Melting point: 360.7 K
Hall symbol: -C 2yc	Mo *K*α radiation λ = 0.71073 Å
*a* = 25.6601 (16) Å	Cell parameters from 1829 reflections
*b* = 5.7624 (3) Å	θ = 2.6–25.8º
*c* = 15.7578 (9) Å	µ = 0.10 mm^−^^1^
β = 92.933 (4)º	*T* = 123 (2) K
*V* = 2327.0 (2) Å^3^	Prismatic, colourless
*Z* = 8	0.50 × 0.50 × 0.50 mm

### Data collection

**Table:**

Bruker KappaAPEXII diffractometer	2672 independent reflections
Radiation source: fine-focus sealed tube	1985 reflections with *I* > 2σ(*I*)
Monochromator: graphite	*R*_int_ = 0.050
*T* = 123(2) K	θ_max_ = 27.5º
0.5° frames in φ and ω scans	θ_min_ = 1.6º
Absorption correction: multi-scan(SADABS; Bruker, 2005)	*h* = −33→33
*T*_min_ = 0.932, *T*_max_ = 0.954	*k* = −7→7
11906 measured reflections	*l* = −20→20

### Refinement

**Table:**

Refinement on *F*^2^	Secondary atom site location: difference Fourier map
Least-squares matrix: full	Hydrogen site location: inferred from neighbouring sites
*R*[*F*^2^ > 2σ(*F*^2^)] = 0.050	H-atom parameters constrained
*wR*(*F*^2^) = 0.106	*w* = 1/[σ^2^(*F*_o_^2^) + (0.0212*P*)^2^ + 2.7981*P*] where *P* = (*F*_o_^2^ + 2*F*_c_^2^)/3
*S* = 1.06	(Δ/σ)_max_ < 0.001
2672 reflections	Δρ_max_ = 0.20 e Å^−^^3^
166 parameters	Δρ_min_ = −0.21 e Å^−^^3^
Primary atom site location: structure-invariant direct methods	Extinction correction: none

### Special details

**Table:**

Geometry. All e.s.d.'s (except the e.s.d. in the dihedral angle between two l.s. planes) are estimated using the full covariance matrix. The cell e.s.d.'s are taken into account individually in the estimation of e.s.d.'s in distances, angles and torsion angles; correlations between e.s.d.'s in cell parameters are only used when they are defined by crystal symmetry. An approximate (isotropic) treatment of cell e.s.d.'s is used for estimating e.s.d.'s involving l.s. planes.
Refinement. Refinement of *F*^2^ against ALL reflections. The weighted *R*-factor *wR* and goodness of fit *S* are based on *F*^2^, conventional *R*-factors *R* are based on *F*, with *F* set to zero for negative *F*^2^. The threshold expression of *F*^2^ > σ(*F*^2^) is used only for calculating *R*-factors(gt) *etc*. and is not relevant to the choice of reflections for refinement. *R*-factors based on *F*^2^ are statistically about twice as large as those based on *F*, and *R*- factors based on ALL data will be even larger.

### Fractional atomic coordinates and isotropic or equivalent isotropic displacement parameters (Å^2^)

**Table:**

	*x*	*y*	*z*	*U*_iso_*/*U*_eq_	
O1	0.33199 (5)	−0.0433 (2)	0.46758 (8)	0.0291 (4)	
O3	0.44741 (5)	0.1331 (2)	0.55663 (8)	0.0317 (4)	
C1	0.35919 (6)	0.0509 (3)	0.40209 (11)	0.0246 (4)	
O2	0.46182 (5)	0.5104 (2)	0.57999 (10)	0.0460 (5)	
C5	0.41061 (7)	0.3418 (3)	0.33969 (11)	0.0296 (5)	
H5	0.4285	0.4860	0.3432	0.036*	
C8	0.34065 (6)	0.3209 (3)	0.54356 (11)	0.0270 (5)	
C14	0.43644 (7)	0.3568 (3)	0.54706 (11)	0.0269 (4)	
C9	0.32151 (7)	0.4651 (4)	0.60572 (11)	0.0340 (5)	
H9	0.3374	0.6117	0.6166	0.041*	
C4	0.40994 (7)	0.2167 (4)	0.26464 (12)	0.0344 (5)	
H4	0.4275	0.2744	0.2174	0.041*	
C2	0.35812 (7)	−0.0765 (3)	0.32741 (11)	0.0296 (5)	
H2	0.3400	−0.2202	0.3236	0.036*	
C12	0.27490 (7)	0.0372 (4)	0.57561 (11)	0.0320 (5)	
H12	0.2591	−0.1099	0.5653	0.038*	
C10	0.27969 (7)	0.3977 (4)	0.65182 (12)	0.0404 (6)	
H10	0.2668	0.4983	0.6936	0.048*	
C11	0.25674 (7)	0.1837 (4)	0.63695 (12)	0.0383 (6)	
H11	0.2282	0.1369	0.6691	0.046*	
C3	0.38359 (7)	0.0071 (4)	0.25876 (12)	0.0336 (5)	
H3	0.3831	−0.0792	0.2074	0.040*	
C6	0.38552 (6)	0.2600 (3)	0.41010 (11)	0.0255 (4)	
C7	0.38629 (7)	0.3930 (3)	0.49288 (11)	0.0265 (5)	
H7	0.3828	0.5622	0.4796	0.032*	
C13	0.31655 (6)	0.1086 (3)	0.52950 (11)	0.0266 (4)	
C15	0.49380 (7)	0.0797 (4)	0.60921 (12)	0.0349 (5)	
H15A	0.4911	0.1505	0.6654	0.052*	
H15B	0.5245	0.1415	0.5825	0.052*	
H15C	0.4972	−0.0889	0.6154	0.052*	

### Atomic displacement parameters (Å^2^)

**Table:**

	*U*^11^	*U*^22^	*U*^33^	*U*^12^	*U*^13^	*U*^23^
O1	0.0329 (7)	0.0252 (7)	0.0294 (7)	0.0015 (5)	0.0027 (5)	−0.0008 (5)
O3	0.0293 (7)	0.0257 (7)	0.0385 (8)	0.0029 (6)	−0.0121 (5)	−0.0011 (6)
C1	0.0234 (8)	0.0252 (10)	0.0251 (9)	0.0051 (7)	−0.0011 (7)	−0.0002 (7)
O2	0.0419 (8)	0.0328 (8)	0.0611 (10)	−0.0017 (7)	−0.0166 (7)	−0.0116 (7)
C5	0.0264 (9)	0.0298 (10)	0.0324 (10)	0.0028 (8)	−0.0027 (7)	0.0057 (8)
C8	0.0260 (9)	0.0296 (10)	0.0248 (9)	0.0088 (7)	−0.0044 (7)	−0.0016 (7)
C14	0.0281 (9)	0.0249 (10)	0.0276 (9)	0.0000 (8)	0.0010 (7)	−0.0043 (8)
C9	0.0329 (10)	0.0396 (12)	0.0284 (10)	0.0133 (9)	−0.0089 (8)	−0.0074 (8)
C4	0.0309 (10)	0.0457 (13)	0.0267 (10)	0.0086 (9)	0.0019 (7)	0.0050 (9)
C2	0.0288 (9)	0.0280 (10)	0.0314 (10)	0.0060 (8)	−0.0048 (7)	−0.0042 (8)
C12	0.0286 (9)	0.0384 (11)	0.0285 (10)	0.0052 (8)	−0.0038 (7)	0.0087 (8)
C10	0.0360 (10)	0.0603 (15)	0.0245 (10)	0.0203 (10)	−0.0028 (8)	−0.0072 (9)
C11	0.0298 (10)	0.0604 (15)	0.0246 (10)	0.0124 (10)	0.0005 (7)	0.0091 (9)
C3	0.0321 (10)	0.0426 (12)	0.0254 (10)	0.0111 (9)	−0.0044 (7)	−0.0061 (8)
C6	0.0248 (8)	0.0244 (10)	0.0267 (9)	0.0050 (7)	−0.0040 (7)	0.0002 (7)
C7	0.0310 (9)	0.0204 (9)	0.0277 (9)	0.0051 (7)	−0.0037 (7)	−0.0015 (7)
C13	0.0279 (9)	0.0303 (10)	0.0210 (9)	0.0082 (8)	−0.0030 (7)	0.0017 (7)
C15	0.0261 (9)	0.0417 (12)	0.0361 (11)	0.0036 (8)	−0.0071 (8)	0.0010 (9)

### Geometric parameters (Å, °)

**Table:**

O1—C13	1.384 (2)	C4—C3	1.385 (3)
O1—C1	1.386 (2)	C4—H4	0.9500
O3—C14	1.326 (2)	C2—C3	1.379 (3)
O3—C15	1.448 (2)	C2—H2	0.9500
C1—C6	1.384 (2)	C12—C11	1.382 (3)
C1—C2	1.386 (2)	C12—C13	1.385 (2)
O2—C14	1.201 (2)	C12—H12	0.9500
C5—C4	1.384 (3)	C10—C11	1.381 (3)
C5—C6	1.393 (2)	C10—H10	0.9500
C5—H5	0.9500	C11—H11	0.9500
C8—C13	1.384 (3)	C3—H3	0.9500
C8—C9	1.393 (2)	C6—C7	1.512 (2)
C8—C7	1.509 (2)	C7—H7	1.0000
C14—C7	1.522 (2)	C15—H15A	0.9800
C9—C10	1.382 (3)	C15—H15B	0.9800
C9—H9	0.9500	C15—H15C	0.9800
			
C13—O1—C1	116.79 (14)	C11—C10—H10	120.1
C14—O3—C15	115.78 (14)	C9—C10—H10	120.1
C6—C1—O1	122.37 (15)	C10—C11—C12	120.52 (18)
C6—C1—C2	121.80 (16)	C10—C11—H11	119.7
O1—C1—C2	115.83 (16)	C12—C11—H11	119.7
C4—C5—C6	121.21 (18)	C2—C3—C4	120.11 (17)
C4—C5—H5	119.4	C2—C3—H3	119.9
C6—C5—H5	119.4	C4—C3—H3	119.9
C13—C8—C9	117.98 (17)	C1—C6—C5	117.73 (16)
C13—C8—C7	120.76 (15)	C1—C6—C7	120.31 (16)
C9—C8—C7	121.26 (17)	C5—C6—C7	121.95 (16)
O2—C14—O3	124.06 (17)	C8—C7—C6	109.91 (15)
O2—C14—C7	124.42 (16)	C8—C7—C14	108.79 (14)
O3—C14—C7	111.45 (15)	C6—C7—C14	112.80 (14)
C10—C9—C8	120.9 (2)	C8—C7—H7	108.4
C10—C9—H9	119.6	C6—C7—H7	108.4
C8—C9—H9	119.6	C14—C7—H7	108.4
C5—C4—C3	119.72 (18)	C8—C13—O1	122.03 (15)
C5—C4—H4	120.1	C8—C13—C12	121.96 (17)
C3—C4—H4	120.1	O1—C13—C12	116.00 (17)
C3—C2—C1	119.42 (18)	O3—C15—H15A	109.5
C3—C2—H2	120.3	O3—C15—H15B	109.5
C1—C2—H2	120.3	H15A—C15—H15B	109.5
C11—C12—C13	118.81 (19)	O3—C15—H15C	109.5
C11—C12—H12	120.6	H15A—C15—H15C	109.5
C13—C12—H12	120.6	H15B—C15—H15C	109.5
C11—C10—C9	119.83 (18)		
			
C13—O1—C1—C6	21.8 (2)	C13—C8—C7—C6	22.5 (2)
C13—O1—C1—C2	−157.71 (15)	C9—C8—C7—C6	−157.52 (16)
C15—O3—C14—O2	−1.3 (3)	C13—C8—C7—C14	−101.48 (18)
C15—O3—C14—C7	−178.43 (14)	C9—C8—C7—C14	78.5 (2)
C13—C8—C9—C10	0.0 (3)	C1—C6—C7—C8	−22.1 (2)
C7—C8—C9—C10	179.99 (16)	C5—C6—C7—C8	157.52 (16)
C6—C5—C4—C3	0.5 (3)	C1—C6—C7—C14	99.48 (19)
C6—C1—C2—C3	−0.5 (3)	C5—C6—C7—C14	−80.9 (2)
O1—C1—C2—C3	179.03 (15)	O2—C14—C7—C8	−105.6 (2)
C8—C9—C10—C11	0.6 (3)	O3—C14—C7—C8	71.48 (18)
C9—C10—C11—C12	−0.7 (3)	O2—C14—C7—C6	132.14 (19)
C13—C12—C11—C10	0.1 (3)	O3—C14—C7—C6	−50.7 (2)
C1—C2—C3—C4	0.0 (3)	C9—C8—C13—O1	178.26 (15)
C5—C4—C3—C2	0.0 (3)	C7—C8—C13—O1	−1.7 (2)
O1—C1—C6—C5	−178.53 (15)	C9—C8—C13—C12	−0.6 (3)
C2—C1—C6—C5	1.0 (2)	C7—C8—C13—C12	179.41 (16)
O1—C1—C6—C7	1.1 (2)	C1—O1—C13—C8	−21.5 (2)
C2—C1—C6—C7	−179.37 (15)	C1—O1—C13—C12	157.43 (15)
C4—C5—C6—C1	−1.0 (2)	C11—C12—C13—C8	0.5 (3)
C4—C5—C6—C7	179.39 (16)	C11—C12—C13—O1	−178.37 (15)

### Hydrogen-bond geometry (Å, °)

**Table:**

*D*—H···*A*	*D*—H	H···*A*	*D*···*A*	*D*—H···*A*
C15—H15C···O2^i^	0.98	2.53	3.407 (3)	149
C3—H3···Cg2^ii^	0.95	2.95	3.668 (2)	133
C11—H11···Cg1^iii^	0.95	3.18	3.825 (2)	127
C15—H15B···Cg1^iv^	0.98	3.06	3.432 (2)	104
C15—H15C···Cg1^iv^	0.98	3.11	3.432 (2)	101

Symmetry codes: (i) *x*, *y*−1, *z*; (ii) *x*, −*y*, *z*−1/2; (iii) −*x*+1/2, −*y*+1/2, −*z*+1; (iv) −*x*+1, −*y*, −*z*+1.

### Table 2 Geometrical parameters (Å, °) of the inter-ring π—π interactions. α is the dihedral angle between planes I and J, CgI is the centroid of plane I and CgJ the centroid of plane J.

**Table:**

CgI	CgJ	Cg···Cg	α	Symmetry position of CgJ
Cg1	Cg2	5.590 (1)	59.44	x,1-y,-1/2+z
Cg1	Cg2	4.944 (1)	24.81	1/2-x,1/2-y,1-z
Cg2	Cg1	4.863 (1)	59.44	x,-y,1/2+z
Cg2	Cg2	3.684 (1)	0.03	1/2-x,1/2-y,1-z

Notes: Cg1 is the centroid of ring C1/C6; Cg2 is the centroid of ring C8/C13.
